# A literature review of Chinese traditional Baduanjin qigong for perimenopausal and postmenopausal symptoms

**DOI:** 10.1097/MD.0000000000040235

**Published:** 2024-11-01

**Authors:** Hong-yan Liu, Ya-ge Luo, Jin Zhang, Yue-han Hu, Han-yu He, Jie Li, Hao-ping Mao, Shu-fei Fu

**Affiliations:** a Chinese Medical College, Tianjin University of Traditional Chinese Medicine, Tianjin, China; b State Key Laboratory of Component-based Chinese Medicine, Tianjin University of Traditional Chinese Medicine, Tianjin, China; c College of Culture and Health Communication, Tianjin University of Traditional Chinese Medicine, Tianjin, China; d Huazhong University of Science and Technology, Wuhan, China; e Key Laboratory of Chinese Formulae, Ministry of Education, Tianjin University of Traditional Chinese Medicine, Tianjin, China.

**Keywords:** Baduanjin, depression, osteoporosis, perimenopausal symptoms, postmenopausal symptoms

## Abstract

This review aimed to evaluate the effectiveness and safety of Baduanjin qigong on perimenopausal and postmenopausal symptoms based on clinical trials. A literature search was conducted in 7 databases up to June 30, 2023. The information of study design and observed indicator based on perimenopausal and postmenopausal stage was extracted. We mainly analyzed the effectiveness, safety and the methodology quality. Thirty-five trials were selected, and 17 original studies were considered good methodological quality. During perimenopause, Baduanjin was mainly to treat mood disorders (63.64%, 14/22), among which 6 (42.86%, 6/14) were depression, 2 (14.29%, 2/14) were depression and anxiety, and 1 (7.14%, 1/14) was anxiety, as well as 5 (35.71%, 5/14) sleep disorders. And the exercise program had a duration of 45 to 50 minutes (57.14%, 8/14), 7 times (71.43%, 10/14) a week. The programs lasting 3 months (42.86%, 6/14), accounted for the highest proportion of the exercise program. In the postmenopausal stage, Baduanjin was used to treat osteoporosis (84.62%, 11/13). From the data available, the program with 2 to 3 times a day (81.82%, 9/11) reported the highest number of significant effects, with a maximum duration of 12 months (55.56%, 5/9), followed by 6 months (33.33%, 3/9). A total of 8 trials mentioned the adverse reactions, but none was related to Baduanjin, and the dropout of participants (1.96%, 57/2912) was also not associated with Baduanjin. There is evidence for positive effects of Baduanjin in addressing perimenopausal mental disorders and postmenopausal osteoporosis, but more research is necessary to clarify best practices and quantify results.

## 1. Introduction

Menopause is associated with vasomotor symptoms,^[1]^ increased risk for osteoporosis,^[2,3]^ increased sexual dysfunction,^[4]^ a significant effect on depression,^[5]^ sleep disturbance^[6,7]^ and plasma lipids and lipoproteins,^[8,9]^ and the resulting hypercholesterolemia may lead to cardiovascular disease^[10]^ and Alzheimer’s disease.^[11]^ All of these have potential implications for quality of life and future health for women.^[12]^ With the extension of human life expectancy, women entering menopause have formed a large population. Currently, 10 million women enter menopause every year in China, and epidemiological surveys in some regions of China showed that the prevalence rate of perimenopausal symptoms was 44.1% to 93.5%.^[13]^ It has been estimated that by 2030, 1.2 billion women worldwide will enter menopause.^[14]^ The World Health Organization has listed improving the quality of life in old age as one of the 3 major themes of promoting health in the 21st century.^[15]^

Hormone therapy was the commonly used method of western medicine to prevent and manage climacteric symptoms for perimenopausal and postmenopausal women. Due to the contraindications and adverse reactions,^[16]^ alternative approaches are important.

**Figure 1.**
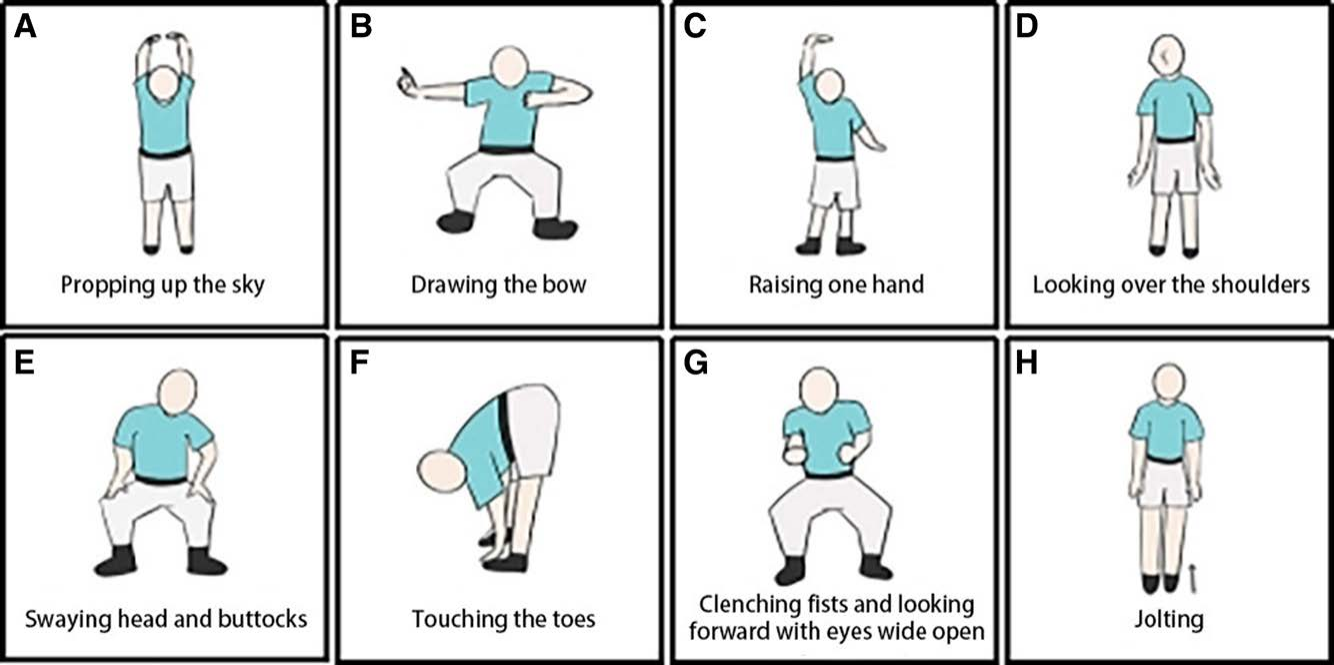
Illustration of Baduanjin. (A) Propping up the hand for regulating triple energizer (San-jiao). (B) Drawing the bow on both sides as if to shoot a vulture. (C) Raising one hand alternately to enhance spleen and stomach function. (D) Looking back the shoulders to treat 5 kinds of strains and 7 impairments. (E) Swaying head and buttocks to subdue the heart fire. (F) Touching the toes to strengthen kidneys and waist. (G) Clenching fists and open eyes widely to enhance vitality. (H) raising up the heels and land on heels repeatedly for 7 times to cure diseases.

Baduanjin is a conventional Chinese fitness exercise which originated in Song Dynasty (almost 800 years ago).^[17]^ It was listed as the 97th sports item officially launched by the Chinese General Administration of Sport in 2003 and has been widely promoted in China.^[5]^ Baduanjin contains 8 simple postures and movements, with slow and fluid movement, mental focus, and controlled breathing.^[18]^ The illustration of Baduanjin is shown in Figure 1 (Designed and drawn by Y.H. in the research team. The software applied for painting was Painting World (Shijiazhuang, China)). Because of its ease of learning and safety,^[19]^ it is accepted by people of all ages.^[20]^ At present, it has been confirmed that Baduanjin can significantly improve symptoms of insomnia,^[21,22]^ depression,^[23]^ and age-related cognitive deterioration,^[24]^ help improve of knee joint proprioception and postural stability, and reduction of pain, stiffness, and functional impairments of old adults with knee osteoarthritis,^[25]^ glycemic control in patients with diabetes,^[26]^ and so on. It is recommended to prevent bone loss^[27]^ and anti-aging in middle-aged women,^[28]^ and to reduce the risk of atherosclerotic cardiovascular disease in patients with prediabetes.^[29]^ Research on the effect of Baduanjin on perimenopausal and postmenopausal symptoms has also been carried out widely^[30,31]^ and has a positive effect on the short-term and long-term influences of menopause. However, these research results have yet to be systematically reviewed. The significance of the study is to comprehensively summarize and evaluate the effectiveness and safety of Baduanjin on perimenopausal and postmenopausal symptoms, providing reference for clinical practice.

## 2. Materials and methods

### 2.1. Search strategy

Two researchers (H.L. and Y.L.) isolated the literature search results. We used PubMed, EBSCO, Embase, Cochrane Library, the China Science and Technology Journal Database (VIP), Wanfang Database (Wanfang), and China National Knowledge Infrastructure to search the literature on treating menopausal symptoms with Baduanjin. The advanced search was conducted using the following terms “Menopause” or “Menopausal Symptom” or “Climacteric” or “Perimenopausal woman” or “Postmenopausal woman” containing “Baduanjin” or “ba duan jin” or “Eight-section Brocade” or “Qigong.” The literature search was up to June 30, 2023, from the establishment of the database. The primary search strategy was shown as followed.

#### 2.1.1. English search strategy

#1 Baduanjin
#2 ba duan jin
#3 Eight-section Brocade
#4 Qigong
#5 (#1 or #2 or #3 or #4)
#6 menopause
#7 climacteric
#8 menopausal symptom
#9 perimenopausal woman
#1 0 postmenopausal woman
#11 (#6 or #7 or #8 or #9 or#10)
#12 (#5 and #11)

#### 2.1.2. Chinese search strategy

((SU=八段锦 AND SU=(绝经+更年期+经断) NOT SU=(鼠+兔+蛋白+细胞+概述+述评+综述)) NOT TI=(研究进展+meta分析)

### 2.2. Inclusion and exclusion criteria

#### 2.2.1. Inclusion criteria

Based on the equity, diversity, and inclusion statement, 3 kinds of clinical trials were included.

Type of studies. Studies such as randomized controlled trials (RCT), non-randomized controlled clinical trials (NRCT), and case series (CS) were included. Types of participants. Studies on perimenopausal and postmenopausal participants were included. Intervention. Studies on Baduanjin as the cardinal intervention were included.

#### 2.2.2. Exclusion criteria

Type of studies. Studies categorized as review, Meta-Analysis, experience summary, clinical practice guideline, and books were excluded. Type of exercise. Studies of comprehensive exercise therapy were excluded. Duplicated publication was excluded.

### 2.3. Data extraction and analysis

Two researchers (H.L. and Y.L.) extracted the data independently. Inconsistencies were resolved through the senior researcher (S.F.). NoteExpress, a document management software, was used to manage the studies obtained from different databases. A Microsoft Access database was established to extract information on the publication of the literature (title, author, journal, time of issue, type of fund), research design (design scheme, number of cases, withdrawal), intervention protocols (frequency and duration), research observation indicators, the safety of the interventions, and other information for statistical analysis.

The methodological quality of included controlled clinical trials was evaluated by the modified Physiotherapy Evidence Database (PEDro) scale^[32]^ (Sydney, Australia), which involves randomization, concealed allocation, similar baseline, blinding of assessor, retention, missing data management, between-group comparison, point measure and measures of variability, isolated Baduanjin intervention, and prior sample size estimation. A sum score ranging from 0 to 10 can be awarded for each study. The descriptive method was used to report adverse reactions.

This study was written in accordance with SANRA guideline.^[33]^

## 3. Results

### 3.1. Description of studies

**Figure 2.**
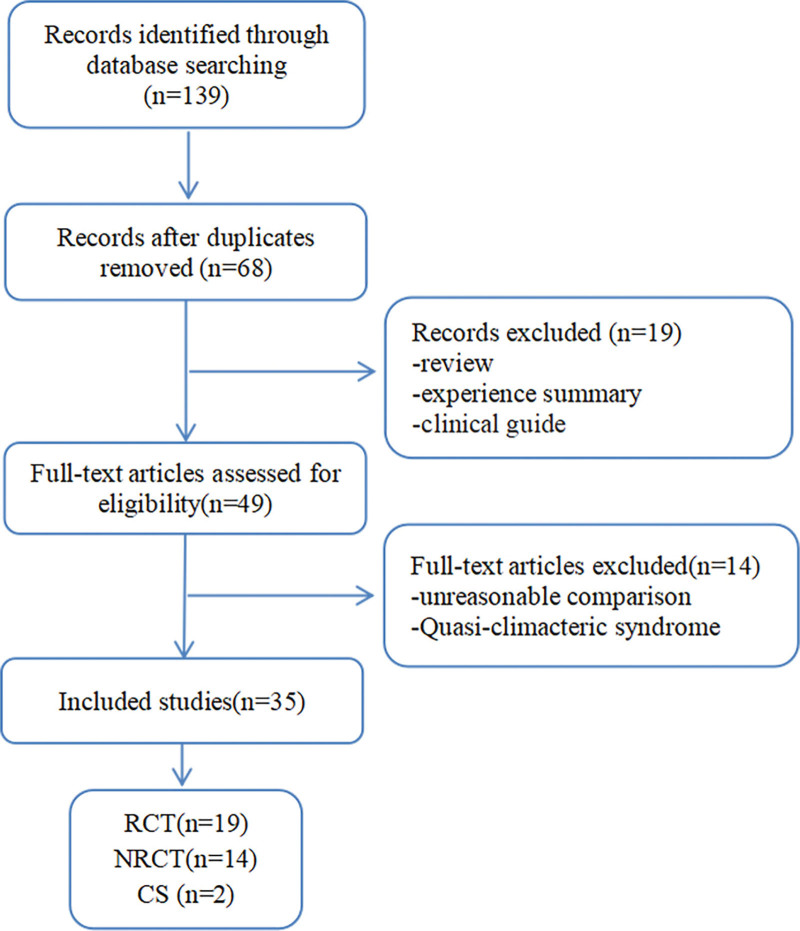
Literature screening flowchart. CS = case series, NRCT = non-randomized controlled clinical trials, RCT = randomized controlled clinical trials.

According to the search strategy, we obtained 139 related articles. After removing of duplicates, 68 records remained. After going through the titles and abstracts, we excluded 19 articles. By reading the full text of the remaining 49 articles, 14 were excluded in which the comparison set of 12 trials did not meet the inclusion criteria, and 2 were quasi-climacteric syndrome caused by Aromatase Inhibitors treatment. Ultimately, 35 articles were included in the present study,^[34–68]^ 33 were in Chinese and 2 in English.^[47,65]^ Among these studies, 19 RCTs, 14 NRCTs (including a cluster-NRCT), and 2 CSs were included, accounting for 54.29% (19/35), 40.0% (14/35), and 5.71% (2/35) of the total number of studies, respectively. The specific screening process is shown in Figure 2.

### 3.2. Characteristics of including studies

The studies involving Baduanjin for treating menopausal symptoms were published between 2009 and 2023. Twenty-nine were academic journal articles, 5 were master’s dissertations, and 1 was a conference article, respectively. All the studies were conducted in China, except for one^[34,65]^ in Spain. The total number of participants was 2912 (with a dropout rate below 10%). Nineteen trials mentioned withdrawal and dropout, of which 7 indicated the reasons for withdrawal. The maximum number of subjects was 198^[47]^ while the minimum was 30.^[40]^ The ages ranged from 40 to 72. The intervention time ranged from 3 weeks to 12 months. Among the 35 studies included, the indication for treatment was postmenopausal osteoporosis, perimenopausal depression, and perimenopausal sleep disorder, accounting for 31.43% (11/35), 17.14% (6/35), and 14.29% (5/35), respectively. A total of 12 studies mentioned the measurement of exercise intensity: 9 measured heart rates while 3 used the Borg Ratings of Perceived Exertion Scale. Of the 35 studies included, 14 were supported by research projects.

The specific characteristics of Baduanjin therapy used for perimenopausal symptoms are shown in Table 1. Those used for postmenopausal symptoms are shown in Table 2.

**Table 1:** Application of Baduanjin in the treatment of perimenopausal symptoms

Included trials	Symptoms	Study design	Sample size (Dropout)	Age (yr)	Intervention Protocol		Outcome index	Inter group differences/ pre-after differences	Adverse reactions/follow-up
					Training duration	Exercise intensity measurement			
Yang 2023	Depression, Anxiety	RCT	80 (0) BJ: 40 CG: 40	BJ: 47.88 ± 2.49 CG: 48.75 ± 2.41	BJ: 4 weeks + drug therapy CG: drug therapy	NM	FEV1, FEV1%, FVC, 6MWD, CAT, SDS, SAS	P1, Pa, Pb < 0.05	NM/NM
Zhang 2021	Depression	RCT	80 (0) BJ: 40 CG: 40	BJ: 48.24 ± 1.19 CG:48.12 ± 1.22	BJ: 7 × 50 min/wk for 3 weeks + drug therapy CG: drug therapy	HR < 120/min	KI, SDS, QLS	P1 < 0.05	NM/NM
Ma 2011	Depression	RCT	145 (0) BJ: 49 WG: 46 CG: 50	BJ:46.89 ± 2.69 WG:47.68 ± 2.56 CG:46.92 ± 2.31	BJ: 5 × 45 min/wk for 3 months WG:5 × 45 min/wk for 3 months CG: no intervention	HR < 100/min	KI KI(II) CES-D	P1 < 0.01 P2 < 0.05 Pa < 0.01 P1, P2 < 0.01	NM/NM
Ma 2011	Depression	RCT	149 (0) BJ: 50 RG: 50 CG: 49	BJ: 46.30 ± 2.60 RG:48.10 ± 2.80 CG:46.50 ± 2.10	BJ: 5 × 45 min/wk for 20 weeks RG: 3 × 45 min/wk for 20 weeks CG: no intervention	HR < 100/min	KI, CES-D	P1 < 0.05 (week 20) Pa < 0.05 (week 10) Pa < 0.05 (week 10/week 20) P3 < 0.05 (week 20)	NM/NM
Suo 2016	Depression	NRCT	60 (0) BJ: 30 CG: 30	BJ: 44–56 CG: 45–55	BJ: 7 × 60 min/wk for 3 months + drug therapy CG: drug therapy	NM	TE, HAMD	P1 < 0.05 P1 < 0.05 Pa < 0.001 Pb > 0.05	NM/6 months
Ma 2010	Depression	NRCT	100 (0) BJ: 50 CG: 50	BJ:49.08 ± 2.76 CG:49.30 ± 2.59	BJ: 7 × 45 min/wk for 3 months CG: no intervention	HR < 100/min	KI, CES-D	P1 < 0.01	NM/NM
Zhou 2011	Depression	CS	30 (0)	BJ: 48.05 ± 2.56	BJ: 7 × 45 min/wk for 6 months	HR < 100/min	KI, CES-D	Pa < 0.01 (month 3) Pa < 0.01 (month 3/month 6)	NM/NM
Wang 2019	Anxiety, depression	RCT	60 (0) BJ: 30 CG: 30	BJ: 49.27 ± 1.25 CG: 49.29 ± 1.28	BJ: 7/week for 3 months + drug therapy CG: drug therapy	NM	TE, NS	P1 < 0.05	NM/NM
Teng 2021	Anxiety	NRCT	60 (0) BJ: 30 CG: 30	BJ: 44.10 ± 3.13 CG: 44.37 ± 3.05	BJ: 7 × 30 min/wk for 90 days + drug therapy + education program CG: drug therapy + education program	NM	KI, HAMA, TE	P1, Pa, Pb < 0.05	No/NM
Jin 2017	Insomnia	RCT	60 (0) BJ: 30 CG: 30	BJ: 49.30 ± 5.10 CG: 49.20 ± 5.50	BJ: 7 × 45 min/wk for 4 weeks + auricular point therapy CG: auricular point therapy	NM	TE	P1 < 0.05	NM/NM
Zhang 2016	Insomnia, Anxiety, Depression	RCT	93 (0) BJ: 47 CG: 46	BJ: 47.90 ± 6.30 CG: 48.40 ± 6.70	BJ: 7 × 45 min/wk for 3 months + education program CG: education program	NM	PSQI, SAS, SDS	P1, Pa < 0.05 Pb > 0.05	NM/NM
Yang 2022	Insomnia	NRCT	60 (0) BJ: 30 CG: 30	BJ: 48.76 ± 5.13 CG: 48.18 ± 5.89	BJ: 7 × 45 min/wk for 4 weeks + auricular point pressing bean CG: auricular point pressing bean	NM	KI, PSQI SF-36, SAS, SDS, TE	P1 < 0.05 (week 2, 4) P1 < 0.05 (week 4)	NM/NM
Lu 2021	Insomnia	NRCT	70 (0) BJ: 35 CG: 35	BJ: 53.50 ± 1.50 CG: 52.20 ± 1.50	BJ: 14 × 30 min/wk for 6 weeks + emotional nursing CG: emotional nursing	NM	TE	P1 < 0.05	NM/NM
Yang 2020	Insomnia	NRCT	80 (0) BJ: 40 CG: 40	BJ: 54.10 ± 1.40 CG: 53.60 ± 1.80	BJ: 7 × 60 min/wk for 6 weeks + emotional nursing CG: emotional nursing	NM	TE, PSQI	P1 < 0.05	NM/NM
Hao 2013	Menopausal symptom cluster	RCT	113 (10) BJ: 55 (6) CG: 58(4)	BJ: 48.90 ± 3.75 CG: 49.60 ± 4.31	BJ: 14 × 45 min/wk for 5 months CG: no intervention	HR < 100/min	KI(II), SF-36(II)	P1, Pa < 0.05	NM/NM
Long 2011	Menopausal symptom cluster	RCT	54 (5) BJ: 27 (2) CG: 27 (3)	BJ: 49.76 ± 3.71 CG: 49.33 ± 3.02	BJ: 13 × 30 min/wk for 12 weeks CG: no intervention	NM	TE KI E₂, FSH, LH	P1 < 0.001 P1, Pa < 0.01 Pb > 0.05 P1, Pa < 0.05 Pb > 0.05	NM/NM
Wang 2017	Menopausal symptom cluster	RCT	60 (0) BJ: 30 CG: 30	BJ: 49.27 ± 1.25 CG: 49.29 ± 1.28	BJ: 7/week for 3 months + drug therapy CG: drug therapy	NM	TE, Satisfaction	P1 < 0.05	NM/NM
Wang 2022	Menopausal symptom cluster	NRCT	69 (6) BJH: 22(1) BJM: 23(2) BJL: 24(3)	BJH: 48.56 ± 2.33 BJM: 48.76 ± 2.33 BJL:49.24 ± 3.61	BJH: 7/week for 12 weeks BJM: 3/week for 12 weeks BJL: 1/week for 12 weeks	RPE Score: 8-14	KI TE, MENQOL E₂	P4, P5 < 0.001 (week 6, 12) Pc, Pd < 0.05 (week 6,12) Pc, Pd < 0.05 P4, P6 < 0.05 (week 6/week12) P4 < 0.05 P5 > 0.05 (week 6) P4, P5 > 0.05 (week 12) Pc, Pd, Pe > 0.05 (week 12)	No/NM
Xue 2018	Functional premature ventricular contraction	RCT	60 (0) BJ: 30 CG: 30	BJ: 50.18 ± 5.97 CG: 49.77 ± 6.36	BJ: 3 × 30 min/wk for 1 month + 5 × 40 min/wk for 1 month + drug therapy CG: drug therapy	RPE Score: 11-13	TE, FFPVC HRV	P1 > 0.05 (month 1) P1 < 0.05 (month 2) P1 < 0.05 (month 1, 2) Pb > 0.05 (month 2)	No/NM
Ma 2011	Functional constipation	NRCT	83 (0) BJ: 43 CG:40	45–55	BJ: 5 × 60 min/wk for 12 weeks CG: no intervention	NM	BSFS, SF-36(II)	P1, Pa < 0.05 Pb > 0.05	NM/NM
Si 2009	Change of Serum Lipid level	Cluster-NRCT	60 (6) BJ: 30 (0) CG: 30 (6)	BJ: 46.20 ± 5.20 CG: 48.50 ± 6.50	BJ: 14 × (10–15) min/wk for 12 weeks CG: no intervention	RPE Score: 11–14	BMI WHR, MDSS MDSS(II) LDL HDL NO	Pa, Pb > 0.05 Pa > 0.05 Pa < 0.05 Pa > 0.05 Pb < 0.05 Pa, Pb > 0.05 Pa, Pb < 0.05	NM/NM
Cheng 2017	Osteoporosis	CS	80 (0)	BJ: 49.00 ± 8.37	BJ: 6 × 30 min/wk for 12 months	NM	QUALEFFO-41, BMD (L1–4) BMD (FN)	Pa < 0.05 Pa > 0.05	NM/NM

6MWD = 6-minute walking distance, BJ = Baduanjin, BJH = Baduanjin (high frequency), BJL = Baduanjin (low frequency), BJM = Baduanjin (medium frequency), BMD = bone mineral density, BMI = body mass index, BSFS = Bristol stool form scale, CAT = COPD assessment test, CES-D = Center for Epidemiological Studies-Depression, CG = control group, CS = Clinical Series, E_2_ = Estradiol, FEV1 = Forced Expiratory Volume in one second, FFPVC = Frequency of Functional Premature Ventricular Contraction, FSH = Follicle Stimulating Hormone, FVC = Forced Vital Capacity, HAMA = Hamilton Anxiety Scale, HAMD = Hamilton Depression Scale, HDL = High Density Lipoprotein Choleslerol, HR = heart rate, HRV = heart rate variability, II = individual item, KI = Kupperman Index, LDL = Low Density Lipoprotein Choleslerol, LH = Luteinizing Hormone, MDSS = Menopausal Disturbance Symptom Scale, MENQOL = Menopause-specific Quality of Life Questionnaire, NM = not mentioned, NO = Nitric Oxide, NRCT = non-randomized Controlled Trials, NS = nursing satisfaction, P1 = Intergroup Difference (BJ-CG), P2 = Intergroup Differences (BJ-WG), P3 = Intergroup Differences (BJ-RG), P4 = Intergroup Differences (BJH-BJL), P5 = Intergroup Differences (BJM-BJL), P6 = Intergroup Differences (BJH-BJM), Pa = Pre-after Differences (BJ), Pb = Pre-after Differences (CG), Pc = Pre-after Differences (BJH), Pd = Pre-after Differences (BJM), Pe = Pre-after Differences (BJL), PSQI = Pittsburgh Sleep Quality Index, QLS = quality of life scale, Qualeffo-41 = Quality of Life Questionnaire of European Foundation for Osteoporosis, RCT = randomized controlled trials, RG = rope skipping group, RPE = Borg Ratings of Perceived Exertion Scale, SAS = Self-rating Anxiety Scale, SDS = Self-rating Depression Scale, SF-36 = the Short Form-36 Health Survey, TE = therapeutic effect, WG = walking group, WHR = Waist-to-Hip Ratio.

**Table 2:** Application of Baduanjin in the treatment of postmenopausal symptoms

Included trials	Symptoms/complication	Study design	Sample size (Dropout)	Age (yr)	Intervention protocol		Outcome Index	Intergroup differences/ pre-after differences	Adverse reactions /follow-up
					Training duration	Exercise intensity measurement			
MDC 2022	Insomnia	RCT	117 (8) BJ: 60 (6) CG: 57 (2)	BJ: 69.70 ± 6.15 CG: 69.75 ± 6.76	BJ: 2 × 60 min/wk for 12 weeks CG: education program	NM	PSQI, HADS	P1, Pa < 0.05	NM/NM
Wang 2021	Osteoporosis	RCT	90 (0) BJ: 45 CG: 45	BJ: 62.38 ± 3.50 CG: 62.35 ± 3.52	BJ: 2 × 30 min/d + tonic diet CG: tonic diet	NM	TE (S + BMD), BMD, VAS	P1 < 0.05	NM/NM
Su 2018	Osteoporosis	RCT	80 (0) BJ: 40 CG: 40	BJ: 58.93 ± 4.01 CG: 59.12 ± 3.88	BJ: 10 × (45–60) min/wk for 6 months + drug therapy CG: drug therapy	HRmax×(70–80%)	TE (S + BMD) VAS, BBS TUG, BMD, BGP, E₂, U-DPD/Cr	P1 < 0.05 P1, Pa, Pb < 0.05	NM/NM
Zhang 2017	Osteoporosis	RCT	74 (2) BJ: 37 (1) CG: 37 (1)	BJ: 53.53 ± 1.42 CG: 53.69 ± 1.14	BJ: 10/wk for 12 months + drug therapy + education program CG: drug therapy + education program	NM	BMD (L2-4) BMD (L-FN) Ca, ALP P	P1, Pa, Pb < 0.05 P1, Pa, Pb > 0.05 P1 > 0.05 Pa, Pb < 0.05 P1, Pa, Pb > 0.05	No/NM
Liu 2015	Osteoporosis	RCT	184 (14) BJ: 48 (2) BJ1: 49 (1) CG: 45 (5) CG1: 42 (6)	50–75	BJ: 3/day for 12 months BJ1: 3/day for 12 months + drug therapy CG: drug therapy CG1: no intervention	NM	VAS BMD (L2-4) Height HSSS	P1, P2 < 0.001 Pa, Pa1 < 0.001 Pa, Pa1, Pb > 0.05	No/NM
Peng 2019	Osteoporosis/Type2 Diabetes Mellitus	RCT	72 (6) BJ: 36 (2) CG: 36 (4)	BJ: 60.88 ± 4.59 CG: 62.31 ± 4.96	BJ: 10/wk for 6 months + drug therapy CG: drug therapy	NM	FBG， 2hPG, HbA1c, BMD (L2-4), BGP, DPD/Cr P, Ca, ALP	Pa, Pb < 0.05 P1 < 0.05 Pa, Pb > 0.05	No/NM
Peng 2018	Osteoporosis/Type2 Diabetes Mellitus	RCT	102 (0) BJ: 51 CG: 51	BJ: 66.88 ± 4.59 CG: 67.31 ± 4.96	BJ: 10/wk for 12 months + drug therapy + education program CG: drug therapy + education program	NM	TE, FO BMD (L2-4, FN) P, Ca BGP, DPD/Cr	P1 < 0.05 P1 < 0.05 Pa, Pb < 0.05 Pa, Pb > 0.05 P1 < 0.05 Pa, Pb < 0.05	No/NM
Cai 2018	Osteoporosis	RCT	60 (0) BJ: 30 CG: 30	BJ: 52.10 ± 4.20 CG: 51.40 ± 4.90	BJ: 10 × 30 min/wk for 12 months + drug therapy CG: drug therapy	NM	BMD (L2-4) P, Ca, ALP QLS	P1 < 0.05 Pa, Pb < 0.05 P1 > 0.05 Pa, Pb > 0.05 P1 < 0.05	NM/NM
Chen 2015	Osteoporosis	NRCT	100 (0) BJ: 50 CG: 50	BJ: 61.20 ± 4.90 CG: 60.80 ± 5.80	BJ: 14/wk + tonic diet CG: tonic diet	NM	TE (S + BMD), VAS	P1 < 0.05	NM/NM
Du 2014	Osteoporosis	NRCT	120 (0) BJ: 40 CG: 40 CG1: 40	61.19 ± 5.50	BJ: 14/wk for 6 months + tonic diet CG: drug therapy CG1: tonic diet	NM	VAS TE (S + BMD) RR	P1, P3 < 0.05 P1, P3 < 0.01 P1, P3 < 0.05	No/NM
Wang 2012	Osteoporosis	NRCT	36 (0) BJ: 18 CG: 18	57.21 ± 3.40	BJ: 10 × 50 min/wk for 20 weeks CG: no intervention	Duration > 5 min (HR > (170-age)/min)	BMD (L-FN, L3, 4) BMD (L-WT, TR, L2) BMC (L-FN, WT, TR, L2, 3, 4) BGP ALP U-HOP, U-Ca, U-HOP/Cr, U-Ca/Cr	P1 < 0.05 P1 > 0.05 P1 < 0.05 P1 < 0.01 P1 > 0.05 P1 < 0.01 P1 > 0.05	NM/NM
Miao 2012	Osteoporosis	NRCT	58 (0) BJ: 12 YJ: 11 WX: 12 LJ: 11 CG: 12	56.12 ± 2.96	BJ, YJ, WX, LJ: 6 × 60 min/wk for 12 months CG: no intervention	HRmax = 120/ min HRmean = 100/min	BMD (DU1/3, DR1/3， L2) P, Ca ALP U-DPD/Cr	Pa < 0.05 Pb > 0.05 P1 > 0.05 P1 < 0.05 P1 < 0.01	NM/NM
Zhu 2014	Early aging	NRCT	42 (0) BJ: 21 CG: 21	BJ: 53.90 ± 4.05 CG: 55.80 ± 4.67	BJ: 5 × 60 min/wk for 6 months CG: no intervention	NM	Weight, BMI, WHR SOD MDA XOD CAT	Pa, Pb > 0.05 (month 3, 6) P1, Pa < 0.05 (month 3) P1, Pa < 0.01 (month 6) Pa < 0.05 (month 3/month 6) P1, Pa < 0.01 (month 3, 6) P1 < 0.05 (month 3, 6) Pa > 0.05 (month 3, 6) P1, Pa > 0.05 (month 3, 6)	NM/NM

2hPG = 2-hour Postprandial Blood Glucose, ALP = Alkaline Phosphatase, BBS = Berg balance scale, BGP = Bone Gla-protein, BJ = Baduanjin, BMC = bone mineral content, BMD = bone mineral density, BMI = body mass Index, CAT = catalase, CG = control group, Cr = creatinine, DPD = deoxypyridinoline, DR1/3 = Distal radius 1/3, DU1/3 = Distal ulna 1/3, E_2_ = Estradiol, FBG = fasting blood glucose, FN = the Femoral Neck, FO = fall occurrence, HADS = The Hospital Anxiety and Depression Scale, HbA1c = Glycosylated Hemoglobin, HOP = hydroxyproline, HR = target heart rate, HSSS = Hospital for Special Surgery Score, L-FN = the Left Femoral Neck, LJ = Liuzijue, L-TR = the Left Trochanter, L-WT = the Left Ward’s triangle, MDA = Malondialdehyde, NM = not mentioned, NRCT = non-randomized controlled trials, P1 = Intergroup Differences (BJ-CG), P2 = Intergroup Differences (BJ1-CG), P3 = Intergroup Differences (BJ-CG1), Pa = Pre-after Differences (BJ), Pa1 = Pre-after Differences (BJ1), Pb = Pre-after Differences (CG), PSQI = Pittsburgh Sleep Quality Index, QLS = quality of life score, RCT = randomized controlled trials, RR = recurrence rate, SOD = superoxidedismutase, TE = therapeutic effect, TUG = time up and go test, U = urine, VAS = visual analogue scale, WHR = waist-to-hip ratio, WX = Wuqinxi, XOD = Xanthineoxidase, YJ = Yijinjing.

### 3.3. Methodological quality

The methodological quality of studies (only involved RCT) was rated according to the PEDro scale (Sydney, Australia), ranging from 5 to 8 points. Of the 19 studies, 17 were considered good methodological quality (6–8 points), while 2 were fair (5 points). Only 3 studies used blinding of assessors, and neither used allocation concealment, leading to the risk of bias. The risk of bias also came from the absence of a priori sample size estimation (94.74%, 18/19) and the application of isolated Baduanjin intervention (68.42%, 13/19). Specific methodological quality is shown in Table 3.

**Table 3:** Methodological quality for randomized clinical trials

Included trials	Item 1	Item 2	Item 3	Item 4	Item 5	Item 6	Item 7	Item 8	Item 9	Item 10	Item 11	Score (Items 2–11)
Yang 2023	√	1	0	1	0	1	1	1	1	0	0	6/10
MDC 2022	√	1	0	1	1	1	0	1	1	1	0	7/10
Zhang 2021	√	1	0	1	0	1	1	1	1	0	0	6/10
Wang 2021	√	1	0	1	0	1	1	1	1	0	0	6/10
Peng 2019	√	1	0	1	0	1	0	1	1	0	0	5/10
Wang 2019	√	1	0	1	0	1	1	1	1	0	0	6/10
Cai 2018	√	1	0	1	0	1	1	1	1	0	0	6/10
Su 2018	√	1	0	1	0	1	1	1	1	0	0	6/10
Xue 2018	√	1	0	1	0	1	1	1	1	0	1	7/10
Peng 2018	√	1	0	1	0	1	1	1	1	0	0	6/10
Jin 2017	√	1	0	1	0	1	1	1	1	0	0	6/10
Zhang 2017	√	1	0	1	0	1	0	1	1	0	0	5/10
Wang 2017	√	1	0	1	0	1	1	1	1	0	0	6/10
Zhang 2016	√	1	0	1	0	1	1	1	1	0	0	6/10
Liu 2015	√	1	0	1	0	1	0	1	1	1	0	6/10
Hao 2013	√	1	0	1	0	1	0	1	1	1	0	6/10
Ma 2011	√	1	0	1	1	1	1	1	1	1	0	8/10
Long 2011	√	1	0	1	0	1	0	1	1	1	0	6/10
Ma 2011	√	1	0	1	1	1	1	1	1	1	0	8/10

Item 1, eligibility criteria specified; Item 2, randomization; Item 3, concealed allocation; Item 4, similar baseline; Item 5, blinding of assessors; Item 6, more than 85% retention; Item 7, missing data management (intention-to-treat analysis); Item 8, between-group comparison; Item 9, point measure and measures of variability; Item 10, isolated Baduanjin intervention; Item 11, prior sample size estimation 1, explicitly described and present in details; 0, absent, inadequately. described, or unclear.

### 3.4. Application of Baduanjin in treating perimenopausal symptoms

In 22 trials on Baduanjin in treating perimenopausal symptoms, there were 11 RCTs, 9 NRCTs (including one cluster-NRCT), and 2 CSs. Of which 6 trials were on depression, 5 on insomnia, 4 on menopausal symptom clusters, 2 on depression and anxiety, and 1 on anxiety. In addition, 3 trials assessed the efficacy of functional ventricular premature beats, functional constipation, and osteoporosis, respectively, and 1 evaluated the effects on blood lipid levels. The specific application of Baduanjin in treating perimenopausal symptoms is shown in Table 1.

Of the 20 trials with control groups, 15 had alternative interventions. Of which, 6 trials^[48,52,57,59,64,68]^ were intervened with drugs, two^[60,61]^ received emotional nursing, two^[51,67]^ received auricular point therapy, one^[49]^ received education program, and one^[62]^ received a combination of drug therapy and education program. At the same time, rope skipping^[38]^ and walking^[39]^ were also used to compare the curative effect with Baduanjin, and one^[66]^ was to compare Baduanjin with different frequencies. Furthermore, the other 5 trials had no intervention in control groups.

In the Baduanjin intervention groups, the intervention time was 3 weeks to 12 months, 3 to 14 times per week, but most (72.73%, 16/22) were daily, totaling 21 to 288 times. Each exercise ranged from 10 to 60 minutes, with most being 45 minutes (36.37%, 8/22), and the total exercise time ranged from 17.5 to 210 hours per week. Four trials reported incomplete information.

Twenty-two studies involved 14 scales. KI (Kupperman index) was usually adopted as a scale to evaluate the perimenopausal symptoms, while occasionally MENQOL (Menopause-Specific Quality of Life) and MDSS (menopausal disturbance symptom scale). The most frequently used instrument for measuring depression and anxiety was CES-D (the Center for Epidemiological studies-depression), SDS (the Self-rating Depression Scale), and SAS (Self-rating Anxiety Scale), followed by HAMD (Hamilton Depression Scale) and HAMA (Hamilton Anxiety Scale). One study evaluated the efficacy of Baduanjin on anxiety based on the satisfaction of nursing care. For the sleep quality measurement, 3 studies adopted PSQI (Pittsburgh sleep quality index), and another two studies were based on symptoms. Three studies adopted SF-36 (short form-36 health survey) to evaluate the quality of life of perimenopausal women. The other 2 studies used Qualeffo-41 (quality of life questionnaire of European foundation for osteoporosis) and QLS (quality of life scale), respectively. Moreover, BSFS (Bristol stool form scale) was used to measure perimenopausal constipation. In addition, there were specific measurements to evaluate osteoporosis, functional ventricular premature beats, and the level of blood lipids.

There were significant differences between Baduanjin and the control group, or(and) before and after the Baduanjin. In addition, 3 studies reported before-and-after differences at different time points during treatment.

### 3.5. Application of Baduanjin in treating postmenopausal symptoms

Of the 13 trials on postmenopausal symptoms, there were 8 RCTs and 5 NRCTs. Eleven were about the effects of Baduanjin on osteoporosis, of which 2 were diabetes mellitus with osteoporosis. Two trials studied Baduanjin on insomnia and free radical levels, respectively. One trial^[47]^ adopted a 2 × 2 factorial design, and the 2 factors were the Baduanjin and medication, each tested on 2 levels. Except for above, 9 control groups received positive interventions, in which 3 control groups^[47,54,56,58]^ were intervened with drugs alone, 3^[44,46,63]^with a tonic diet alone, one^[41,42,45,65]^ with education program alone, and 2^[53,55]^ with a combination of drug therapy and education program. The specific application of Baduanjin in treating postmenopausal symptoms is shown in Table 2.

For the Baduanjin intervention groups, 6 trials did not report the time spent on each exercise, 2 did not illustrate the duration of the intervention, 1 only mentioned workouts twice a day, and did not clarify the time spent on each exercise and the intervention duration, only 6 were comprehensive. From the data available in trials on osteoporosis, the intervention duration ranged from 20 weeks to 12 months, with 6 to 21 times a week. A typical exercise lasted between 30 to 60 minutes, and the total workout time was from 166.67 to 288 hours. Of the 13 articles, 3 mentioned exercise intensity and took heart rate as the measurement index, and 1 mentioned that each exercise should ensure that the heart rate exceeds the target heart rate for more than 5 minutes.

In all trials on osteoporosis, BMD (bone mineral density) was adopted as an outcome index, followed by biochemical indexes of bone metabolism. At the same time, the severity of pain is often measured using VAS (visual analog scale). In addition, tests, 3′UG (3-feet up-and-go test), OLS (one-leg stance), and TUG (time up-and-go test), were conducted to measure the balance and motor performance in 2 trials. Some scales, such as BBS (Berg Balance Scale), QLS (Quality of Life Scale), and HSSS (Hospital for Special Surgery Score), were used in the trials on postmenopausal osteoporosis. Apart from that, HADS (Hospital Anxiety and Depression Scale) was adopted to evaluate the magnitude of anxiety symptoms and depression due to insomnia, while free radicals, Serum MDA (Malondialdehyde), XOD (Xanthineoxidase), SOD (Superoxide dismutase), and CAT (Catalase), for postmenopausal women aging.

There were significant differences between Baduanjin and the control group, or(and) between before and after the exercise. In addition, one study reported pre-after differences at different time points during treatment.

### 3.6. Adverse reactions analysis

Eight trials mentioned adverse reaction monitoring, including indicators related to Baduanjin, such as an electrocardiogram (25.0%, 2/8), 4 vital signs (25.0%, 2/8), and knee synovitis (12.50%, 1/8). As well as liver function (62.50%, 5/8), renal function (50.0%, 4/8), blood routine (25.0%, 2/8), and urine routine (12.50%, 1/8). No adverse reactions were reported. Other trials did not specify whether adverse reactions occurred. Of the 2912 participants in this report, 57 were lost. Either because of failure to comply with the intervention protocol (26.31%, 15/57), other diseases (35.09%, 20/57), or for unspecified reasons (38.60%, 22/57). None mentioned dropout because of the adverse reactions of Baduanjin.

## 4. Discussion

A comprehensive systematic review of research studies published before June 30, 2023, was performed to review the evidence of Baduanjin on perimenopausal and postmenopausal conditions. A total of 35 original research studies were retrieved and analyzed.

Different duration of Baduanjin is in accordance with the perimenopausal diseases. Osteoporosis (5/11) takes the longest time, 12 months, followed by depression and anxiety, 3 to 6 months (7/9), menopausal symptoms, functional constipation, and early aging, most of the duration is 3 months (5/6), the duration for insomnia is 4 to 6 weeks (4/5), and for functional ventricular premature beats is the shortest, only 1 month.

Thirteen trials involving Baduanjin were used in perimenopausal mental health disorders (14/22). Baduanjin was a separate intervention for depressive symptom. When the condition met the diagnostic criteria for depression, Baduanjin was often combined with medication. For perimenopausal sleep disorders, the intervention of Baduanjin achieved positive results in combination with health education (including emotional nursing) or auricular point pressing bean. Moreover, the exercise program with a frequency of 45 to 50 minutes (61.54%, 8/13, time taken for practicing 2 sets of Baduanjin including warm-up part, intermediate rest and cooling-down part) 7 times (76.92%, 10/13) a week and a program duration of 3 months (46.15%, 6/13), reported the highest number of significant effects. Studies confirmed that not only regulated the body, but more importantly, regulated the mental state, which can exercise the coordination and flexibility of the nervous system, relieve brain fatigue, and regulate emotions.^[69]^ Some studies also showed that Baduanjin effectively increased the levels of adiponectin levels^[70]^ and monoamine^[71]^ and regulated the dysregulated expression of lncRNA, mRNA, and circRNA,^[72]^ which potentially contributed to reducing depression and anxiety in menopausal women, the sleep disorders were relieved. The quality of life may improve accordingly. In addition, 3 trials^[36,48,62]^ showed that the use of Baduanjin in the treatment of depression and anxiety should be based on TCM theory.

In eleven trials on postmenopausal women osteoporosis, supplementing calcium and vitamin D (54.55%, 6/11) is essential to prevent and treat osteoporosis. The addition of Baduanjin can promote calcium absorption, and the blood calcium in the body moves into bone, promoting the proliferation of bone cells, increasing bone density, and delaying bone loss.^[56]^ From the data available, 2 to 3 times a day (81.82%, 9/11) reported the highest number of significant effects, with a maximum duration of 12 months (55.56%, 5/9), followed by 6 months (33.33%, 3/9) and a minimum one of 20 weeks (11.11%, 1/9). It shows that postmenopausal women should increase the amount of exercise within their scope to achieve better prevention and control effects. Eleven trials achieved consistently positive results in improving lumbar BMD, increasing Bone Gla Protein (BGP) and reducing the ratio of Deoxypyridinoline (DPD) to Creatinine (Cr) in urine, reducing pain, improving balance ability, and quality of life are thus improved. It has been commonly accepted that physical exercise provides an osteogenic stimulus^[73]^ and promotes osteogenic differentiation^[74]^ by regulating functions of differentiated cells in the skeletal tissue.^[75]^ It may also be associated with correcting epigenetic alterations, restoring Nrf2 loss, and improving femoral bone mass and trabecular microarchitecture.^[76]^ Uncovering such information was complicated by the enormous variation in the duration and intensity of exercise programs.

As a common exercise, Baduanjin often combines with other therapies to play comprehensive therapeutic effects in diseases.^[77,78]^ Such research design cannot directly reflect the role played by the individual application of Baduanjin. This study provides a summary of clinical studies that demonstrate the individual effects of Baduanjin for the treatment of perimenopausal and postmenopausal symptoms, which not only supplemented the lack of relevant literature but also made its application more targeted by analyzing the application of Baduanjin in different stages of menopause. Although encouraging results were found in this review, some limitations must be recognized. First, the trials had some risk of bias, which came from the lack of concealed allocation, blinding, and prior sample size estimation in most original studies. Second, the comparability between the Baduanjin intervention protocol could have been better. The same disease requires a unified exercise program. In osteoporosis research, most trials did not propose a definite exercise program. Even when interventions were available, they varied widely, affecting the accuracy of assessments. Third, because of the limited number of studies on the effects of Baduanjin on functional constipation, functional premature ventricular contraction, anxiety, and blood lipid levels, it remains unclear whether these outcomes are incidental.

## 5. Conclusion

There is evidence for positive effects of Baduanjin in addressing perimenopausal mental health disorders and postmenopausal osteoporosis. Though some studies showed that Baduanjin had efficacy on menopausal symptom cluster, functional ventricular premature beats, functional constipation, change of serum lipid level, and on postmenopausal insomnia and early aging, more research is necessary to clarify best practice and quantify results.

## Acknowledgments

Thanks to the teachers in the library of Tianjin University of Traditional Chinese Medicine for their help in searching for the full text of the included literature.

## Author contributions

**Conceptualization:** Shu-fei Fu.

**Data curation:** Ya-ge Luo, Han-yu He, Jie Li.

**Formal analysis:** Hong-yan Liu.

**Methodology:** Hong-yan Liu, Han-yu He, Shu-fei Fu.

**Software:** Jie Li.

**Supervision:** Shu-fei Fu.

**Visualization:** Yue-han Hu.

**Writing – original draft:** Hong-yan Liu.

**Writing – review & editing:** Hong-yan Liu, Jin Zhang, Hao-ping Mao, Shu-fei Fu.
